# A Review of Transbuccal Fentanyl Use in the Emergency Department

**DOI:** 10.1155/2012/768796

**Published:** 2012-03-20

**Authors:** Annette O. Arthur, Peyton Holder

**Affiliations:** Department of Emergency Medicine, University of Oklahoma School of Community Medicine, 1145 South Utica Avenue, Suite 600, Tulsa, OK 74104, USA

## Abstract

Patients with severe, painful injuries and illnesses treated in the emergency department are commonly administered opioid medications. Intravenous administration provides the most rapid onset of pain relief and is readily titrated. Fentanyl, administered intravenously, is well documented as an effective medication for pain management in the emergency department. It is preferred in many settings due to its minimal hemodynamic effects, as compared to other commonly used opioids. However, not all patients require intravenous access. These patients are given orally administered pain medications. The oral route is effective at minimizing pain but has a much slower onset of action when compared to the intravenous route. As an alternative to the slower onset of action seen with oral opioids, this paper discusses the use of fentanyl buccal tablet for pain management in the emergency department. Fentanyl buccal tablets are readily absorbed, with a bioavailability of approximately 65%, and have a more rapid onset of action than achieved with traditional oral opioids used in the emergency department.

## 1. Introduction

The relief of acute pain secondary to minor orthopedic injuries is often delayed and inadequate in the emergency department [1, 2]. This issue, referred to as oligoanalgesia, has been demonstrated in both the USA and abroad. The frequent delay in analgesia delivery is best illustrated by a UK study that revealed a 3-hour 56-minute mean time to receipt of analgesia for patients with moderate pain. On average, there was a 1-hour 8-minute delay between analgesia prescription and administration [2]. In addition, for cases in which oral analgesics are prescribed, the relief of pain is even further delayed by their relatively slow onset.

Oral NSAIDS and opioids are commonly used in patients with minor orthopedic injuries, but with several disadvantages. The many risks of NSAIDs have been well described, and NSAID therapy has consequences specific to orthopedic injuries (nonunion) [3]. Although oral opioids do not have the same risk profile as NSAIDs, both of these options have a much slower onset than intravenous opioids.

Intravenous opioids are often chosen for their rapid onset among other benefits. While intravenous access is typically easy to obtain in the emergency department, it may be otherwise unnecessary in many patients with minor orthopedic injuries. In these instances, obtaining intravenous access not only exposes the patient to increased discomfort but further delays medication administration. 

The time to pain relief in patients with minor orthopedic injuries could be significantly reduced by an alternative analgesic. This alternative would ideally have a rapid onset and be administered in a simple fashion via a nonparenteral route. With these goals in mind, the authors propose an off-label indication for fentanyl buccal tablet (FBT) as a novel form of analgesia in the emergency department for patients with isolated minor orthopedic injuries.

## 2. Transbuccal Fentanyl Pharmacokinetics and Pharmacodynamics [4]

Fentanyl is an intravenous analgesic commonly used in the emergency department (ED). The intravenous dosage form exhibits linear pharmacokinetics. Another dosage form of fentanyl, the FBT, also reflects linear pharmacokinetics, in a nearly dose-proportional manner, over the 100 mcg to 800 mcg dose range. FBT is absorbed readily with a bioavailability of 65%. The buccal mucosa is the initial location of absorption, with peak plasma concentrations occurring within an hour of administration. Transmucosal absorption accounts for approximately 50% of the total administered dose. Swallowing contributes to the other 50% of the total dose absorption, over a prolonged period, in the gastrointestinal tract. As a comparison, FBT exhibits an approximately 28% greater bioavailability over the oral transmucosal fentanyl citrate (OTFC; “lozenges”), FBT 65% and OTFC 47%. This difference is likely due to the FBT exhibiting approximately 55% more of the total dose absorbed transmucosally than with OTFC. This difference also results in the inability to substitute at an equal dose these two transmucosal forms of fentanyl. In a study comparing OTFC with oral oxycodone for pediatric outpatient wound care, the investigators found that transmucosal administration of opioids produced similar sedation and analgesia to other opioid delivery methods [5]. Increased bioavailability, more rapid onset of analgesia, and faster time to peak plasma levels are also seen with the transmucosal opioid delivery systems. The transmucosal fentanyls display an onset of analgesia action in 5 to 15 minutes with a peak effect seen in 15 to 30 minutes. The fentanyl systemic exposure is not apparently affected by the length of time an FBT tablet takes to disintegrate fully in the buccal area after administration. When the FBT is compared to oxycodone immediate release (IR) for breakthrough pain in chronic pain patients the primary measure of efficacy, the level of pain intensity difference at 15 minutes, significantly favored FBT over the oxycodone [6]. The adverse events reported in this study were typical of opioid pain medication and were similar between the 2 treatment groups, FBT and oxycodone IR.

Fentanyl, including transmucosal forms, is highly lipophilic. It is also greatly bound, 80–85%, to plasma protein. Alpha-1-acid glycoprotein is the main binding protein, but fentanyl also binds to albumin and lipoproteins. The mean steady-state volume of distribution is 25.4 L/kg. Fentanyl is metabolized by the cytochrome P450 3A4 system in the liver to norfentanyl, a pharmacologically inactive metabolite. Inactive metabolites account for more than 90% of the elimination of fentanyl, with less than 7% of the total dose administered, excreted unchanged in the urine.

## 3. Existing Evidence for Fentanyl Buccal Tablet

Intravenous fentanyl is frequently used in acute care and has a proven track record. A rapid onset, short duration of action, good response to opiate antagonists, and minimal hemodynamic effects make fentanyl a very desirable option in the acute care setting [7, 8]. While the intravenous form is commonly used, alternative administration routes include the fentanyl buccal tablet, the fentanyl buccal soluble film, oral transmucosal fentanyl (lozenges), intranasal fentanyl, and nebulized fentanyl. 

Although oral transmucosal fentanyl has been frequently used in the emergency department for pediatric procedural sedation, the transbuccal forms have been shown to have both greater bioavailability and a more rapid onset [9–11]. Intranasal and nebulized fentanyl are also viable options but intranasal fentanyl has primarily been studied in pediatrics and nebulized fentanyl lacks the simple method of delivery that FBT has to offer [12, 13]. In contrast, the fentanyl buccal tablet is simple to administer while still providing a rapid onset. In healthy volunteers, a single 400 mcg dose of FBT has been shown to have a mean time to maximum plasma concentration of 52.2 minutes [14]. To put this in perspective, a single 5 mg swallowed dose of immediate release oxycodone has a mean time to maximum plasma concentration of 1.4 hours (a difference of 31.8 minutes) [15]. 

Use of the fentanyl buccal tablet has traditionally been reserved for breakthrough pain in cancer patients. This is the only Federal Drug Administration (FDA) approved indication for this dosage form of fentanyl. This population has been well studied, and transbuccal fentanyl has been shown to have a more rapid onset when compared to immediate release oxycodone in these patients [6]. Transbuccal fentanyl can be used in the emergency department to fill the previously described void between oral and intravenous analgesia. Although current emergency department research focuses on the fentanyl buccal tablet form, it should be acknowledged that the fentanyl buccal soluble film may also be an excellent choice [11, 16]. 

Currently, the literature regarding fentanyl buccal tablet use in the ED setting consists of the Fentanyl Administered Intraorally for Rapid Treatment of Orthopedic Pain in the ED (FAIRTOP) trial [17]. In this double-blind randomized controlled trial, 60 participants were given either a fentanyl buccal tablet (100 mcg) with a swallowed placebo or an oxycodone/acetaminophen (5/325 mg) tablet swallowed with a nonopioid transbuccal “placebo.” A lansoprazole 15 mg transbuccal tablet was used as the nonopioid transbuccal “placebo.” With a two-unit drop in numeric pain scale (0 to 10) as the primary endpoint, the median time to endpoint was 10 minutes in the fentanyl buccal tablet group versus 35 minutes in the oxycodone/acetaminophen group (*P* = .0001). The reported adverse effects were minimal in this study. There was one episode of vomiting in the oxycodone/acetaminophen group and 13% of patients overall experienced nausea (27% of the oxycodone/acetaminophen group and 0% of the fentanyl buccal tablet group, *P* = .005). Only one case of nausea was described as being greater than slight nausea. In addition, some subjects described feelings such as “woozy” or “dizzy,” but this had an approximately equal frequency between the groups and is not unusual with the use of narcotics. Vital signs were monitored and no other adverse effects were noted.

The investigators of the FAIRTOP trial acknowledged both its limitations and the need for further investigation. First, while the sample size was large enough to demonstrate a significant difference in time to primary endpoint, reduction in pain level, the low sample size provided limited information concerning side effects. Secondly, there was some question as to whether an equianalgesic dose of fentanyl and oxycodone/acetaminophen was used. Lastly, although there was no known source for selection bias, it is a concern due to convenience sampling. Despite these limitations, the results are very promising. That being said, with the limited data in the published literature, further investigation is needed to more clearly define both safety and efficacy and to guide decision making prior to widespread transbuccal fentanyl use in the ED setting.

## 4. Future Research

The FAIRTOP II trial aims to further define the benefits of FBT. As a secondary objective, this new trial intends to provide more information regarding side effect rates and a comparison of the time to onset between a 200 mcg dose of FBT to 2, oxycodone/APAP 5 mg/325 mg tablets. There is a need for future studies to examine the effects of doses greater than 100 mcg and 200 mcg in the prehospital and ED population, for patients experiencing severe pain.
